# Lorenzo Tomatis and primary prevention of environmental cancer

**DOI:** 10.1186/1476-069X-10-S1-S14

**Published:** 2011-04-05

**Authors:** Ronald L Melnick, James Huff

**Affiliations:** 1 Ron Melnick Consulting, LLC, 111 Roundtree Rd, Chapel Hill, NC 25514, USA; 2 National Institute of Environmental Health Sciences, Research Triangle Park, NC 27709, USA

## Abstract

The leading 20^th^ century proponent for primary prevention of environmental cancer was Dr. Lorenzo Tomatis, the former Director of the International Agency for Research on Cancer and founder of the IARC Monographs program. This paper is dedicated to the memory of Dr. Tomatis – eminent scientist, scholar, teacher, humanitarian, and public health champion - and includes many perspectives that he promoted throughout his career, with original quotations from some of his scientific writings on primary prevention of environmental cancer. Any attempt by us to simply summarize his views would only detract from the power and logic of his language.

*“Cancer still remains a mainly lethal disease. Primary prevention remains the most relevant approach to reduce mortality through a reduction in incidence”*[*1*].

## Obstacles in implementing primary prevention strategies for environmental cancer

The principle behind primary prevention of environmental cancer is simple: prevent disease occurrence by avoiding or maximally reducing human exposure to agents recognized as human carcinogens or to agents for which there is experimental evidence of carcinogenicity. While this strategy seems logical from a public health perspective, its implementation has unfortunately encountered numerous obstacles. While people overwhelmingly prefer policies that prevent disease occurrence over treatments that prevent or delay disease progression, federal health science budgets overwhelmingly support research aimed at the development of therapeutic treatments compared to the identification of agents that are hazardous to human health.

For all public health decisions and actions there are benefits and costs. The avoidance of human exposure to environmental carcinogens provides an important health benefit to the exposed public and future generations; however, it may also impose additional financial costs to those who produce the product or release the carcinogenic agent into the environment. Because of uncertainties in predicting the exact level of risk due to exposure to agents that are carcinogenic in laboratory animals, those who bear the costs associated with reduction or elimination of human exposure have frequently exaggerated the uncertainties and lobbied vigorously to deny the validity of experimental carcinogenicity data and its utility in assessing human cancer risk. “Risks may be ‘low’ and ‘hypothetical’ only to those who are unaffected or do not share equally the benefits of reduced exposure. Disease prevention strategies cannot rely on agenda-driven proclamations made by some individuals regarding what constitutes miniscule hypothetical risks” [1].

“Primary prevention of cancer has stumbled from the very beginning because of the interference of powerful economic interest which perceived that any data indicating a possible cancer risk after exposure to industrial chemicals jeopardizes their profits, the protection of which being more important than the protection of human health” [2]. “Because of the determination of powerful economic interests to maintain the level of their profits at all costs, no international agreement yet exists to ban the production and use of asbestos worldwide”[3]. The arguments against primary prevention are not very different from those used recently to deny the existence of global climate change and the role of human activities. Ignoring true risks or delaying the implementation of preventive measures can lead to catastrophic consequences for current and future generations. Use of tobacco is a prime example in which the recognition and implementation of prevention strategies were unduly delayed.

Unfortunately, “dismissing animal carcinogenicity findings would lead to human cancer cases as the only means of demonstrating carcinogenicity of environmental agents” [1]. “The experimental approach to carcinogenicity can ascertain and predict potential cancer risks to humans in time for primary prevention to be successful. As epidemiological studies cannot give early warning of a cancer risk, [accepting epidemiological data] as the only reliable evidence of a carcinogenic effect in humans….is equivalent to accepting that a potential hazardous effect of an environmental agent can be assessed only *a posteriori*, after the agent has had time to cause its harmful effects” [4]. “What appeared to have been forgotten, and in any case disregarded, was that results obtained in long-term carcinogenicity tests had in several instances preceded observations in humans and could have permitted the adoption of measures for reducing or avoiding exposure to carcinogens in the working environment” [5].

Tomatis had frequently expressed concerns about the limitations of epidemiological data for public health decisions. “Inadequacy of epidemiological evidence of carcinogenicity [is in part related to] the extreme caution with which some epidemiologists assess evidence for risk for fear of being accused of creating false-positive results” [3]. “Absent or inadequate epidemiological data cannot be considered equivalent to a negative finding and cannot be considered more relevant for public health than positive experimental findings”[6]. Inadequate epidemiological data or false-negative results can be hazardous to public health, especially in the presence of positive experimental data.

Arguments have been made that doses used in animal studies cannot be extrapolated to human exposures or that carcinogens identified in the workplace are not relevant to environmental exposures. “The labeling of certain carcinogenic chemicals as occupational carcinogens was often interpreted as indicating that the working environment was the only place in which their carcinogenic activity would be revealed; however, they [occupational carcinogens] do not cease to be carcinogenic when present at lower concentrations in the general environment. A typical example is asbestos. [Similarly], tobacco smoke does not cease to be carcinogenic when it is inhaled passively at concentrations that are considerably lower than those actively inhaled” [5]. “The differences between cancers that occur as a consequence of occupational exposure and other cancers are not only their preventability but, more importantly their social unacceptability” [7]. More importantly, Tomatis realized that “exposure levels that could seem as sufficiently low when based on single agents may actually not be safe in the context of the many other concomitant carcinogenic and mutagenic exposures” [8] and “health effects at environmental exposure levels, especially during critical stages of development, have not been fully characterized. This is an important and active area of research that cannot be dismissed by simple denial”[1] .

## Ethical issues and inequalities in environmental cancer prevention

Tomatis often spoke out against inequalities in cancer risks and ethical aspects of environmental disease prevention. “Occupational risks in developing countries are becoming a serious problem, largely as the consequence of the transferring of hazardous industries from industrialized countries where certain industries are now judged unacceptable because of the risks for health and the environment, to developing countries, where adequate legislation protecting the workers and the environment does not yet exist” [9]. “Some chemical compounds were recognized as carcinogens in some countries and not in others, and even where they have been recognized as carcinogenic, the permitted or accepted concentrations varied considerably from country to country, as if their carcinogenicity could disappear or change at certain borders” [3]. “There is no justification for, and it is profoundly unethical to omit, delay, or hide information that may be relevant to the protection of health” [10].

“Attributing most cancer cases to lifestyle, which is interpreted as being related to free personal choice, unduly amplifies the individual’s responsibility, diverts attention from the lack of commitment of health authorities, and obscures the etiological role of other risk factors” [3]. “The assumption that all behavioral choices are free choices does not reflect the actual situation. Individuals cannot really choose the socioeconomic situation in which to be born or their genetic background, and most workers cannot choose to avoid working in hazardous industries or occupations” [6]. In addition to his undeniable quest for primary prevention, Tomatis was a stalwart champion of basic and equal human rights; yet, he came to the vexing realization that “in spite of the many attempts made at various periods of human history to arrive at an equalitarian society by reducing differences between the rich and the poor and by redistributing wealth, social inequalities have not disappeared” [11].

## The need to apply the precautionary principle for environmental carcinogens

Lastly, Tomatis was a strong proponent of the precautionary principle. In spite of uncertainties in estimating cancer risks from environmental exposure to agents that are carcinogenic in laboratory animals or in occupationally exposed workers, arguments such as “people are not rats or mice” or that “doses used in animal studies or occupational exposures are much higher than exposures to the general population” do not counter the fact that the agent under consideration is a carcinogen. Recommendations to delay primary prevention practices until additional data are available do not provide reassurance or health protection to exposed populations. “In the absence of absolute certainty, rarely if ever reached in biology, it is essential to adopt an attitude of responsible caution, in line with the principles of primary prevention, the only one that may prevent unlimited experimentation on the entire human species” [4]. “Primary prevention has the double ethical privilege of intervening for the purpose of avoiding damage to health for the present and future generations ……and of protecting all individuals without the potential for discrimination on socioeconomic grounds” [8].

“It is only prudent to pursue public health measures that are likely to reduce the risk of preventable cancers related to environmental chemicals. The occurrence of large numbers of avoidable cancer cases and associated deaths is a circumstance that society should seek to reduce. Protection of public health is a goal that society should always pursue” [1]. No truer or finer words on the rationale and support for primary prevention have ever been written.

Lorenzo Tomatis [1929 – 2007], a renaissance man in the ultimate sense, promoted the linking of medicine to humanitarianism, science to practicality, long-term bioassays to primary prevention, history to literature, and altruism to environmental and social justice. In most of his topical papers he would be sure to mention the value of primary prevention and the need for more attention to social justice. These were recurring and significant themes in his research, mentoring, teaching, and public health advocacy. His collective and eclectic contributions in cancer causes and prevention are without peer.

## Competing interests

The authors declare that they have no competing financial or non-financial interests.
